# The Interplay Between Sleep and Mental Health: A Genetic Perspective

**DOI:** 10.1016/j.biopsych.2025.06.024

**Published:** 2025-07-05

**Authors:** Nataliia Kozhemiako, Carol Mita, Georgia M. Panagiotaropoulou, Katie JS Lewis, Tamar Sofer, Shaun M. Purcell

**Affiliations:** 1 Brigham and Women’s Hospital, Harvard Medical School; 2 Countway Library, Harvard Medical School; 3 Department of Psychiatry and Psychotherapy, Charité – Universitätsmedizin Berlin; 4 Stanley Center for Psychiatric Research, Broad Institute of Harvard and MIT, Cambridge, Massachusetts, USA; 5 Massachusetts General Hospital, Boston, MA, USA; 6 Centre for Neuropsychiatric Genetics and Genomics, Cardiff University, UK; 7 Division of Psychological Medicine and Clinical Neurosciences, School of Medicine, Cardiff University, UK; 8 Cardiovascular Institute, Beth Israel Deaconess Medical Center, Harvard Medical School, Boston, MA, USA; 9 Department of Biostatistics, Harvard T.H. Chan School of Public Health, Boston, MA, USA; 10 Division of Sleep Medicine, Brigham and Women’s Hospital, Boston, MA, USA

## Abstract

Although many facets of sleep, including subjective, behavioral and neurophysiological features, are closely linked with psychiatric disorders, the natures of these relationships are generally unclear. A given alteration in sleep could reflect a cause (that may mediate genetic risk), consequence, symptom, trigger, epiphenomenon due to shared determinants, or some combination of these. Genetic approaches can in principle be informative: i) by identifying specific genetic influences on disease mediated by or shared with sleep, which could help the search for biological mechanisms and therapeutic targets; ii) by providing evidence for causality, which could suggest interventions for modifiable sleep traits. Here, we summarize recent human quantitative and molecular genetic studies on sleep and psychiatric disease, including twin and genome-wide association studies. Despite evidence for shared heritability across many domains, notably depression and insomnia, the field is in its early stages and faces significant challenges: i) putative causal effects are small, phenotypically non-specific, not resolved to specific gene pathways and often bidirectional; ii) most current discovery cohorts are demographically biased and do not capture profound age-related changes in sleep and its genetic architecture; iii) group-level analyses ignore patient-to-patient heterogeneity, including the presence or absence of specific sleep alterations; iv) a paucity of objective, brain-based data in genetically-informative samples hampers making connections with sleep neurophysiology. Nonetheless, as ever-growing genetic tools and resources still hold great potential for translational bridges between basic model-systems, human epidemiology and personalized clinical care, genetic approaches will still likely be needed to reveal sleep’s roles in maintaining mental health.

## Introduction

Sleep is linked to mental health, with many neuropsychiatric conditions manifesting comorbid sleep disturbances(1,2). While sleep disturbances may result from secondary factors such as medication side effects, they could also be intrinsic to mental health pathophysiology(3), such that sleep physiology shares or mediates genetic risk for disease. Sleep issues can precede the onset of disorders(4), influence their progression and exacerbate symptom severity(5–7). Consequently, genetic studies of sleep and psychiatric disease are broadly motivated by the premise that a better understanding of their common genetic architecture may have implications for disease prediction, diagnosis and treatment.

This review summarizes recent evidence from human studies applying diverse methodologies to explore the genetic connections between sleep and mental health. One immediate challenge arises from the various, conceptually distinct perspectives employed in sleep research, from subjective report (e.g., sleep quality and duration, chronotype), to clinical disorder (e.g., insomnia, sleep apnea), to objective measurement (e.g. multi-day sleep-wake rhythms from actigraphy), to millisecond-level quantifications of sleep neurophysiology from electroencephalography (EEG). While evidence of heritability exists across all these measures (8–11), they reflect fundamentally different aspects of sleep.

**Figure 1:**
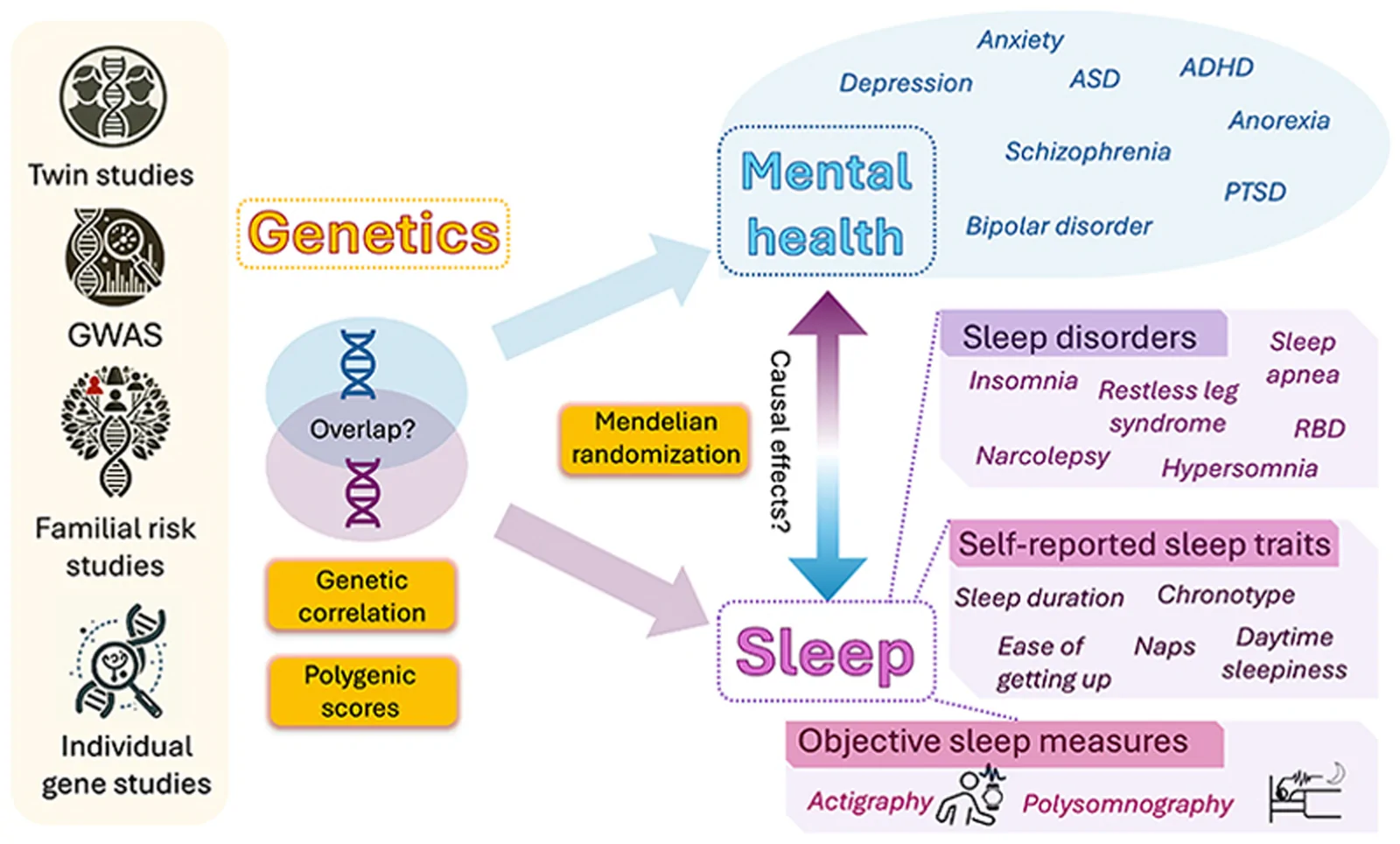
Schematic illustration of the scope and main questions of the review. GWAS – genome-wide association study, ASD – autism spectrum disorder, ADHD – attention-deficit/hyperactivity disorder, PTSD – posttraumatic stress disorder, RBD – rapid eye movement behavior disorder. Description of the different types of genetic studies and approaches shown in the figure is provided in the Supplementary information S1.

We first review studies of genetic correlations between sleep and mental health, based on twin and genome-wide association studies (GWAS), followed by polygenic scores (PGS), which estimate individuals’ genetic risks/propensities (Figure 1). We summarize Mendelian randomization (MR) studies aiming to establish causal bases for associations, and research on specific gene variants. Overall, this review underscores the complex interplay between sleep and mental health as well as the challenges of human genetic epidemiologic approaches.

### Shared genetics between sleep and mental health traits from twin studies

Twin studies quantify genetic and environmental influences on a trait as well as the extent to which shared genes account for the covariance between traits (genetic correlation r_A_, for *additive* effects). For more details see ref. (12) and Supplemental material. Depression and anxiety symptoms have been reported most frequently in relation to sleep(13), typically in population cohorts (Table 1). In children (3-4 years), genetic correlations between parent-reported sleep problems and anxiety symptoms (either at age 3-4 and 7) were small(14). Although a significant genetic correlation (r_A_=0.64) between parent-reported sleep problems and depression symptoms was reported in children aged 8(15), the same sleep measure did not show a significant r_A_ with depression symptoms at age 10, despite being phenotypically associated(16). In adolescent twins, significant genetic overlap was found between sleep quality and anxiety and depression symptoms(17), with similar results in young adult twins for sleep disturbance and depression (r_A_=0.68) or anxiety (r_A_=0.58) symptoms(18). Likewise, significant genetic correlations were reported between sleep quality and depression symptoms (r_A_=0.61) in 18-65 year olds(19). However, in older adults (mean age 68) multiple sleep metrics (including polysomnography and actigraphy) showed only limited shared genetics with depression symptoms, albeit based on a relatively small sample of male twins (20). These results suggest an age-dependent genetic architecture linking sleep and mental health, consistent with the marked changes in sleep across the lifespan.

Insomnia – a core diagnostic criterion for mood disorders including major depressive disorder (MDD) – has also been genetically linked with depression (r_A_=0.73) and anxiety (r_A_=0.59) symptoms in adolescents(17), with similar findings in large twin studies of young adults(21,22) and adults(23,24).

Other commonly measured sleep traits in twin studies include chronotype (morningness-eveningness preference), sleep apnea symptoms and daytime sleepiness. For example, depression symptoms have shown genetic overlap with chronotype (r_A_=-0.21, higher risk in evening types)(25) and sleep apnea symptoms (r_A_=0.6)(26). Results for daytime sleepiness further suggest age-dependent effects: a significant genetic correlation with depressive symptoms in older adults (r_A_=0.21, even controlling for factors such as snoring and metabolic disease)(27) was not detected in adolescents(28). Twin studies of sleep and other psychiatric traits are more limited (**Supplementary Material Section 2**).

Overall, twin studies suggest a significant genetic overlap between sleep and mental health, particularly depression, though the extent of this overlap may vary with age.

### Genome-wide association studies (GWAS)

**Figure 2:**
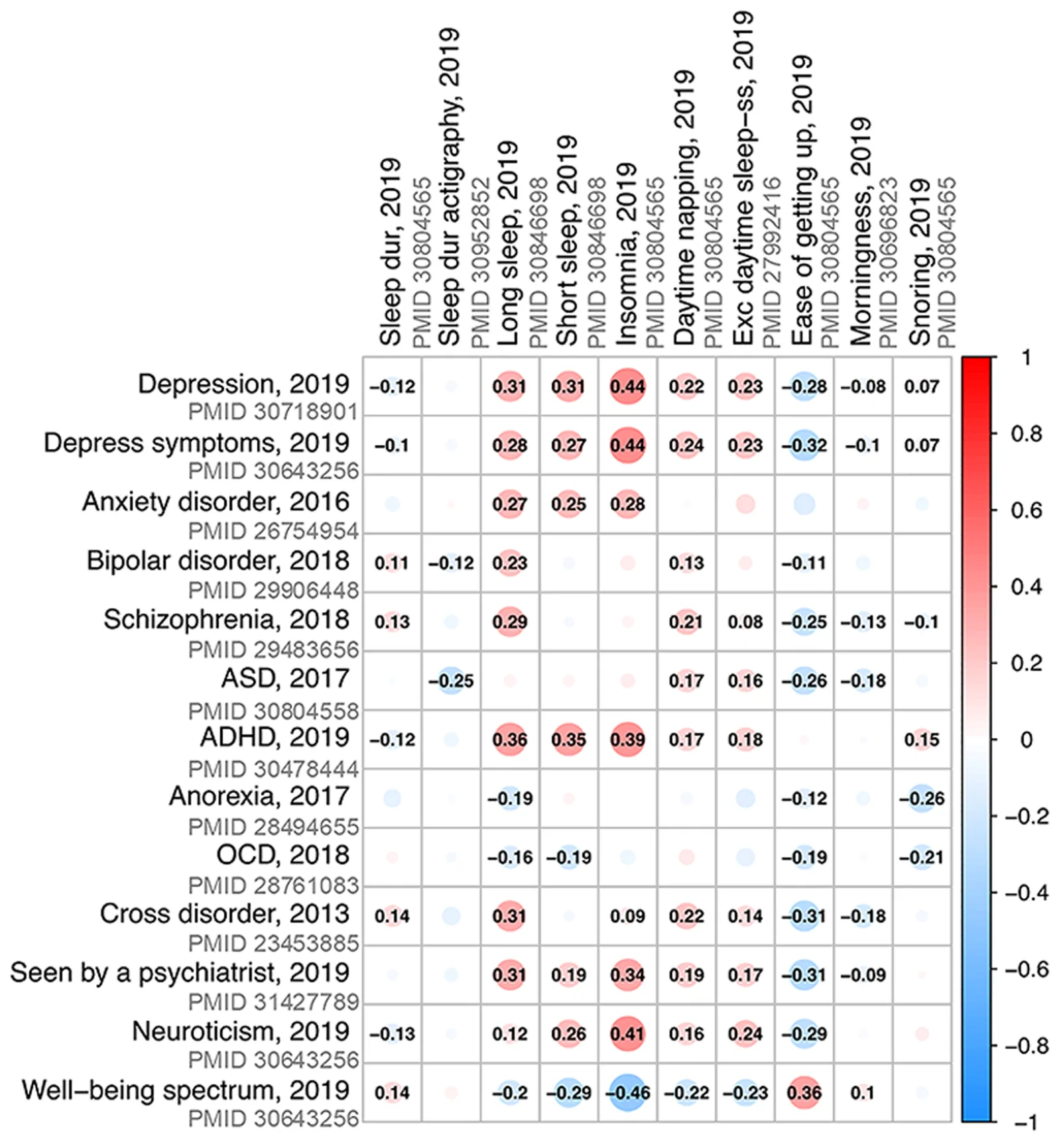
Genetic correlation between sleep phenotypes and traits relevant for mental health from https://atlas.ctglab.nl/multiGWAS. Correlation coefficients are plotted for pairs with p-value < 0.05 after the FDR correction.

Large GWAS can identify specific allelic variants with pleiotropic (shared) effects on sleep and mental health traits (29). Analogous to twin studies, they can also be used to estimate genetic correlations based on the measured aggregate genetic variance (often called r_G_), but with the advantage that the sleep and mental health phenotypes do not need to be measured in the same individuals. Genetic correlations accessible through the GWAS Atlas resource(30) point to significant sharing among sleep traits – including sleep duration, chronotype, naps, daytime sleepiness, ease of getting up, snoring, and insomnia – and mental health phenotypes (Figure 2). The largest genetic correlation was between insomnia and an index of global well-being (r_G_=-0.46). Other sleep phenotypes (long duration, daytime sleepiness, and ease of getting up) displayed multiple significant genetic correlations across a range of mental health related traits. Among neuropsychiatric disorders, depression showed the strongest genetic correlations, particularly for insomnia (r_G_=0.44). A more recent GWAS (593,724 cases, 1,771,286 controls) reported stronger genetic correlations with insomnia, r_G_=0.65 for MDD and r_G_=0.57 for depressive symptoms(31) and also identified genetic correlations between insomnia and other psychiatric conditions, including ADHD, schizophrenia (SCZ), and bipolar disorder (BD) (Table 1).

Several recent studies have systematically compared the genetic architecture of sleep and psychiatric traits. Jia et al. reanalyzed genetic correlations for seven sleep traits – sleep duration, long sleep, short sleep, insomnia, chronotype, daytime napping, and excessive daytime sleepiness – and MDD, BD, and SCZ, identifying significant genetic correlations in 17 of 21 pairs(32). Further analysis identified 443 pleiotropic genes across these trait pairs, with enriched expression in brain tissue in pathways related to synaptic structure/function and neurogenesis. A similar study assessed genetic correlations between six sleep traits – insomnia, short sleep duration, long sleep duration, daytime napping, daytime sleepiness, and being a morning person – and 24 brain disorders, finding that psychiatric disorders had the highest percentage (66%, 48 out of 72 trait pairs) of significant genetic correlations, with the strongest between PTSD and short sleep duration (r_G_=0.44) or insomnia (r_G_=0.43)(33).

Given the well-established and substantial genetic overlap among neuropsychiatric disorders, genetic links to sleep traits are expected to be shared across disorders. Indeed, a cross-disorder GWAS involving eight conditions – anorexia nervosa, ADHD, ASD, BD, MDD, obsessive-compulsive disorder, SCZ, and Tourette syndrome – reported significant genetic correlations with insomnia, daytime sleepiness, and chronotype(34). Further GWAS have introduced new sleep phenotypes, including composite sleep-health scores, nightmares, and sleep medication prescription (Table 1).

Overall, patterns of genetic correlations identified through GWAS broadly confirm the twin studies. GWAS are more informative for heritable outcomes with lower prevalences including SCZ and BD, which are not well captured in population-based studies. Nonetheless, large-scale sleep GWAS are typically constrained to blunt phenotypic measures. For example, one recent study posited that the distinct phenotype of sleep inertia (difficulty getting up in the morning, a marker of circadian misalignment) may better account for links between chronotype and psychiatric disorders, rather than diurnal preference *per se*(35), underscoring the need for greater nuance in measuring sleep.

### PGS-based prediction of genetic risks and propensities

Beyond estimating population parameters such as heritability and genetic correlation, GWAS-derived polygenic scores (PGS) can predict the genetic component of a phenotype(36). PGS studies in unaffected individuals are conceptually related to family-based, unaffected-relative designs and broadly recapitulate results from genetic correlation analyses. For example, a sleep-health composite was inversely associated with PGS for MDD and anxiety in the UK Biobank(37). Conversely, a sleep-health PGS – based on similar UK Biobank information – was associated with mental health traits including depression, anxiety, PTSD and substance use disorder in an independent biobank(38). With respect for other diseases (including ADHD, BD, SCZ and ASD), the data are more mixed (see **Supplementary Material Section S1)**. It is also unclear whether sleep phenotypes observed in psychiatric populations necessarily reflect the same underlying constructs as in general populations (**Supplemental Material Section S4**).

Given the heterogeneity of psychiatric disorders, the risk of diagnostic misclassification, and the limited effectiveness of current treatments, using sleep-based PGS for patient stratification holds potential. For example, in the All of Us cohort, an insomnia PGS was associated with a higher odds of treatment-resistant depression compared to treatment-responsive cases, with those in the top 20th percentile of insomnia PGS showing faster progression to treatment-resistant depression(39). In addition, a study on sleep deprivation for treating depression symptoms found that patients who did not respond to the therapy had significantly higher depression PGS compared to those who did respond(40).

#### Objective measures of sleep

PGS-based studies have been extended to include a range of objective sleep measures. Actigraphy measures were associated with an MDD PGS in the UK Biobank, revealing lower sleep efficiency, more nighttime wakefulness, frequent naps, and higher bedtime variability with higher PGS(41). Conversely, a PGS of low relative amplitude of daily activity – an actigraphy metric often linked to less robust circadian rhythms – was associated with MDD, mood instability and neuroticism(42). Beyond the UK Biobank, PGS and family-based studies have included more detailed characterizations of sleep. For example, polysomnography studies on individuals with a family history of depression have reported altered sleep inter-channel coherence(43,44) and reduced spindle activity(45). Higher REM density and increased sigma power (12-15 Hz, often used as a proxy for sleep spindle activity) during non-rapid eye movement (NREM) sleep was reported for healthy first-degree relatives of MDD or BD patients(46,47). However, sleep EEG studies are limited by small sample sizes.

One area with more robust linkages is the reduction of sleep spindles – markers of NREM sleep – in patients with SCZ. Healthy first-degree relatives of SCZ patients showed reduced sigma power(48), lower fast spindle density(49), and decreased integrated spindle activity(50). *CACNA1I*, which encodes a voltage-gated calcium channel and is expressed in thalamic neurons responsible for spindle generation, is one potential link(51). First degree SCZ relatives also exhibited reduced slow oscillation (SO) amplitude, a signature of deep sleep(50,52). Not all reports are straightforwardly consistent with genetic risk being directly mediated via sleep-dependent processes, however. One study found increased SO-spindle coupling consistency in first-degree relatives of SCZ patients, but no differences in spindle or SO metrics individually(53). Two small PGS-based studies in healthy individuals reported that higher SCZ PGS were associated with greater spindle density, opposite to the direction expected from case-control and familial risk studies (54,55).

Overall, these studies suggest that genetic predisposition to certain mental illnesses is often accompanied by various sleep alterations. However, strong conclusions remain elusive due to variability in sample sizes, methodology, explored sleep features and a lack of replication.

### GWAS and Mendelian randomization

Beyond establishing a genetic correlation, a more pressing question regarding sleep and mental health relates to the causal nature of the associations, which in turn could inform interventions and treatments that modify putatively causal factors. By itself, a genetic correlation between *X* and *Y* does not distinguish between a) *X* causing *Y*, b) *Y* causing *X*, c) bidirectional causality or d) both *X* and *Y* resulting from heritable upstream factors. GWAS data can in principle investigate causal relationships via Mendelian Randomization (MR), which assumes that, as genetic variants are randomly inherited, they can serve as unbiased instrumental variables (correlates of an exposure assumed to affect the outcome only via the exposure)(56).

Earlier reviews of the MR literature on sleep and psychiatric disorders concluded that genetic predispositions to several sleep traits contribute to the risk of disorders(57,58). A plethora of studies have since emerged (Table 2). For example, a) insomnia was reported to be linked to an increased risk of depression, anxiety, BD, ADHD and PTSD; b) daytime napping increased the risk for depression, BD, SCZ, ADHD, ASD, but appeared protective for anorexia; c) morningness was associated with a reduced risk of anxiety and SCZ but increased the odds of anorexia; d) longer sleep duration increased the risk for depression and SCZ. Additionally, these disorders also causally affect sleep and circadian rhythms. For example, whereas short sleep duration(33), insomnia (33,59–63), obstructive sleep apnea (OSA)(64–68) and daytime napping(33,59,62) have been reported to be causal factors for MDD, MDD has also been reported as causal for those sleep traits(32,33,63,65,68,69). However, a different study applying an alternative latent causal variable (LCV) approach to detect putative causal relationships between seven sleep traits and 1,527 other phenotypes came to the different conclusion that most sleep variables were downstream consequences of disease phenotypes and not vice versa(70), suggesting that methodological details may impact the interpretation across many of these studies.

While numerous MR studies suggest potential bidirectional causal links between sleep traits and mental health, MR analyses can be susceptible to various forms of bias from unmet assumptions in general(71), or specific to sleep(72). While MR analyses are now implemented in standard software packages and can be easily automated, a lack of sufficient attention to the details can threaten the validity (73). Furthermore, most studies use a two-sample framework with overlapping GWAS data, limiting their independence. To strengthen causal claims, integrating multiple evidence sources through triangulation is essential(74).

### Specific genetic variants with pleiotropic effects on sleep and mental health

Beyond quantitative genetics, uncovering genetic links between sleep and mental health requires identifying specific gene variants with pleiotropic effects, followed by molecular and functional analyses. To this end, one promising approach is studying sleep disturbances in syndromes with known genetic causes to reveal how impaired genes influence sleep-regulating circuits. Patients carrying a 22q11.2 deletion – a genomic region critical to neurodevelopment and strongly linked to increased rates of SCZ and ASD – exhibit disproportionately high rates of sleep disruption compared to the general population(75). Similarly, 80% of individuals with Phelan-McDermid Syndrome or 22q13.3 deletion syndrome – a disorder caused by chromosome 22 aberrations resulting in defective *SHANK3* gene function and implicated in ASD and SCZ – experience sleep and circadian rhythm issues(76). *SHANK3* plays a critical role in synaptic signaling, with its protein concentrations correlating with melatonin fluctuations in the mouse hippocampus(77). *SHANK3* deficiency also alters EEG spectral power during sleep in mice(78). Numerous other rare neurodevelopmental genetic disorders exhibit similar patterns: a study examining 19 syndromes and six sleep disorder types concluded that sleep disturbances are prevalent across almost all these conditions(79). Another study demonstrated that children with neurodevelopmental genetic conditions have higher risk of insomnia and other sleep issues(80). Furthermore, a recent analysis identified over 100 overlapping genes associated with sleep, epileptic encephalopathies and developmental delays, implicating genes primarily regulating serotonin, dopamine, GABA and endocannabinoid signaling pathways(81).

In terms of GWAS-detected common variants for sleep traits, there are scant examples of unambiguously associated alleles localized to specific genetic mechanisms that might offer inroads into shared neurobiology. In contrast, the molecular genetics of the circadian system has been more extensively delineated. Given alterations in sleep/wake rhythms are observed in many psychiatric traits, studies have investigated the hypothesis that genetic variants in the circadian system may drive risk for various mental health conditions(82). The core clock genes – including *CLOCK, BMAL1, CRY, RORA, PER1, PER2, PER3* – regulate biological rhythms, and common variants in these genes have been identified in large-scale GWAS of chronotype(83). Although a host of smaller-scale candidate gene studies have reported associations with psychiatric outcomes(84), including mood disorders(85,86), BD in particular(87,88), SCZ(89), ADHD(90) and addiction(91), there is relatively little consensus in findings and few core circadian genes have emerged from large-scale psychiatric GWASs(92). Other approaches, for example looking at rare structural variants in ASD have shown enrichment of more broadly-defined sets of circadian genes in ASD, suggesting these effects are independent of sleep disruptions(93).

### Conclusions and future directions

This review summarizes research driven by two overarching, albeit still distal, goals: 1) to detect shared molecular pathways implicating overlapping biological mechanisms between sleep and psychiatric health, potentially leading to new treatments for both, and 2) to identify sleep aspects that mediate genetic risk for psychiatric disease and could serve as therapeutic targets if modifiable.

Twin and genetic correlation studies suggest shared genetic bases between depression and numerous sleep phenotypes, along with bidirectional causal relationships between them. Congruent with the broad sharing of genetic factors across psychiatric conditions, similar overlaps are reported for anxiety, SCZ, BD, ADHD, ASD, anorexia and PTSD, though causal effects remain inconsistent. However, beyond circadian research and insights from rare genetic syndromes, the genetic epidemiology of sleep is still in its infancy. Advancing this field to meet the goals outlined above will require overcoming several challenges.

#### Conceptualizing the role of sleep on disease risk

Much of the work presented here is fundamentally data-driven, with a scope and focus circumscribed by currently available public measures and methods. However, this approach can sometimes overlook complex conceptual issues of sleep-mental health relationships. For example, altered sleep may be a cause and/or consequence of mental illness, but it could also represent a moderating factor that triggers or unmasks pre-existing genetic risk. While twin and GWAS studies have largely focused on genetic *correlation* and *overlap*, more studies that view disrupted sleep as a putative “environmental/behavioral exposure” within a gene-environment *interaction* framework (here, a gene-by-sleep interaction) may be useful.

#### Dependent definitions of exposures and outcomes

A further conceptual issue is the overlap between sleep phenotype and psychiatric diagnoses. In some cases, sleep disturbances are not just putative cause, consequence or modifier of genetic risk for psychiatric disease – they are embedded in the diagnostic criteria for that disease. For example, DSM-5 defines a major depressive episode by symptoms such as daily insomnia or hypersomnia and daytime fatigue with at least five of nine symptoms necessary for diagnosis(94). Likewise, “decreased need for sleep” during manic and hypomanic episodes is among the DSM-5 symptoms of BD. Consequently, mentioned sleep problems cannot be fully separated as independent risk factors or outcomes for these psychiatric disorders because they are wrapped into their very definition. As the result, MDD or BD PGS will *obligatorily* capture individual differences in sleep to some extent, making the explanatory value of sleep as a “genetic cause or correlation” less clear, or even circular.

#### Disease heterogeneity

Many genetic studies rely on diagnosis-based PGS which do not capture symptom-level heterogeneity among patients with the same diagnosis. For instance, while many MDD patients have insomnia, some have hypersomnia, others both or neither. Whether such heterogeneity reflects only a superficial, stochastic expression of the same underlying disease (a.k.a. “DSM got it right”) or whether it indexes more etiologically distinct disease subtypes will typically be an unresolved question. Therefore, population-level aggregate inferences about sleep and mental health (or other medical traits) may lead only to limited insights, if based only on such relatively blunt clinical heuristics.

#### Age-dependent genetic architecture of sleep

Reliance on a few large cohorts adds the further issue of population-dependency: the most relevant causal links may vary by sociodemographic factors, including age and sex, as well as other clinical factors. Almost all aspects of sleep show marked age-dependent changes, and the relative contribution of genetic influence on a trait may shift across the lifespan. For example, sleep duration heritability varied from 17% in infancy, to 69% in adolescence to 42-45% in adulthood (9). Critically, the genes influencing a trait during childhood may no longer be relevant for the same trait later in life, and vice versa(95). Indeed, a study of over 10,000 children found no significant genetic correlation between adult and childhood sleep duration; further childhood sleep duration did not show significant genetic correlations with psychiatric diseases including BD, SCZ and MDD(96). Likewise, given significant age-related changes in sleep architecture and higher prevalence of sleep disorders (e.g. OSA) in older individuals, the genetic determinants of sleep in older cohorts (e.g. UK Biobank, ages 40-69) may show little or no correspondence to the variability among younger individuals, in which the incidence of many psychiatric diseases including SCZ, BD and MDD is highest. Such factors may obfuscate the interpretation of nominally significant causal results, and lead to null findings. This echoes the twin literature, which noted age-dependencies in genetic overlap across child development, further underscoring the centrality of a developmental perspective to the highly dynamic expression of fixed genotypes over the lifespan.

#### Granularity of sleep measures in large population cohorts

While modern genetic methods require large sample sizes, this often limits the granularity of sleep-related phenotypes. The core of the literature above focuses on a handful of metrics – insomnia, sleep duration, diurnal preference, and daytime sleepiness – primarily reflecting the choice of questions included in the UK Biobank survey. Although the inclusion of actigraphy in a subset of the UK Biobank is a welcome exception, there is a notable gap in research utilizing brain-based objective sleep measures, particularly sleep EEG. As a result, the genetic overlap between mental health traits and more precise metrics of sleep neurophysiology – including those already phenotypically linked to disease – remains largely unknown.

#### Statistical power of PGS-based studies

PGS-based analyses use the predicted value of the genetic component of a phenotype rather than the measured phenotype itself. This approach can be attractive for several reasons: it allows investigation of associations between sleep and mental illness in samples where sleep is not directly measured, and is free from common confounds including medication effects. However, even when a PGS explains substantial phenotypic variance, and there is a sizable genetic overlap between the phenotype and outcome, it is typically not demonstrated that reported PGS analyses are statistically well-powered, when applied to relatively small samples (*N*=100-1000). For analyses stratified by diagnostic status, power may be even further impacted.

#### Prioritization by effect sizes

Psychiatric diseases are generally highly polygenic with many small genetic effects and substantial pleiotropy among diagnostic categories. The bivariate relationships between sleep and psychiatric disorders may be similarly polygenic and diffuse. While the current MR literature points to many putatively causal and often bidirectional links, increasing sample sizes may ultimately reveal something more akin to a ‘fully-connected’ network of effects, in which almost every feasible arrow is declared statistically causal, albeit of trivial effect. Under this scenario, it will be critical to focus on a)plausible effect sizes, b) the potential modifiability of exposures and c) the eventual etiologically-oriented reclassification of disease, that manifests a sparser but stronger set of causal relations.

#### The modifiability of sleep traits

In principle, sleep-related behavior and sleep-dependent neurobiology are amenable to therapeutic modulation. Sleep was highlighted in a recent meta-review on “lifestyle factors” and their potential for modification to prevent or treat mental disorders(97). Still, others have noted that significant challenges, including inconsistent protocols, limiting the ability to draw definitive conclusions or conduct meta-analyses for most interventions(98). Nevertheless, cognitive behavioral therapy for insomnia was reported to significantly reduce depression severity in patients diagnosed with insomnia(99). Bright light therapy is promising circadian-based intervention to normalize sleep/wake rhythms, demonstrating medium to large improvements in depressive symptoms in BD patients(98). Others have investigated pharmacological approaches to modifying sleep neurophysiology, for example using eszopiclone, a nonbenzodiazepine sedative hypnotic, to modulate sleep spindle activity in SCZ patients to target cognitive impairments(100,101). Beyond behavioral interventions and pharmacological genetic targets shared between sleep and psychiatric conditions(32,81), neuromodulation-based approaches have been pursued, to therapeutically alter sleep-dependent neurophysiology, for example via closed-loop auditory stimulation(102). Overall, while various modifications are possible, it remains unclear which will be most effective in different clinical contexts and when they should be implemented – with respect to both developmental stage and disease onset – for the greatest impact.

#### Future directions

Research on the genetic architecture of sleep and its role in psychiatric disorders is still emerging. Key areas for future work include: 1) integrating high-dimensional, multivariate objective measures of sleep – particularly those capturing brain activity – with advanced feature extraction methods and robust statistical corrections for multiple testing. These measures should be applied to deeply phenotyped psychiatric cohorts with genetic data and ideally including high-risk individuals at ages relevant to disease onset, with a longitudinal component to capture the dynamic interplay between sleep, psychiatric health, cognition, and other traits; 2) sleep predictors (genetically-based or directly measured) to quantify patient-to-patient heterogeneity rather than case/control diagnostic status, with the goal of predicting or monitoring clinically relevant outcomes including treatment response; 3) comparative characterization of sleep neurophysiological and behavioral phenotypes measured in animal models with their human counterparts, including maturational trajectories, to support a robust translational bridge for specific gene studies paired with advances in the basic molecular genetic characterization of sleep circuitry and psychiatric disease.

## Figures and Tables

**Table 1:** Genetic overlap between neuropsychiatric conditions and sleep traits based on twin studies and GWAS. n.s. – non-significant results as defined in an original report.

Disorder	Genetic overlap based on twin studies	Genetic correlation based on GWAS
Depression or depressive symptoms	r_A_ =0.64 with parent-reported sleep problems in children at 8 years (15), but not significant (r_A_ =0.11) at 10 (16) r_A_ =0.68 with sleep disturbance in adults (18) r_A_ =0.61 with self-reported sleep quality in adults (19) n.s. r_A_ with objective and self- reported sleep metrics in older adults r_A_ =0.7-0.8 with insomnia in adolescents and young adults (17) (21,22) r_A_ =-0.21 with eveningness(25) r_A_ =0.6 with OSA (26) r_A_ =0.21 with daytime sleepiness in older adults(27), but n.s. in adolescents (28)	r_G_=0.44-65 with insomnia (30–33) r_G_=-0.08 with being a morning person (32,33) r_G_=0.17-0.22 with daytime nap (32,33,103) r_G_=0.17 with excessive daytime sleepiness (32,33) r_G_=0.27-0.49 with long sleep duration, r_G_=0.22-0.43 with short sleep duration, r_G_=-0.11 – -0.12 with continuous sleep duration (32,33,104) r_G_=-0.37 – 0.4 with sleep health scores (105,106) r_G_=0.5 with probable bruxism (107) r_G_=0.55 with sleep medication prescription (108) r_G_=0.56 with nightmares (109) r_G_=0.34-0.35 with RLS (110) r_G_=0.27 with difficulty awakening (35)
Anxiety	r*A* =0.58 with sleep disturbance in adults (18) r*A* =0.59 with insomnia (17) n.s r_A_ with daytime sleepiness in adolescents (28)	r_G_=0.4 with insomnia (33) r_G_=-0.08 with being a morning person (33) r_G_=0.15 with daytime nap (33) r_G_=0.16 with excessive daytime sleepiness (33) r_G_=0.29 with long sleep duration, r_G_=0.25 with short sleep duration (33) r_G_=0.43 with probable bruxism (107) r_G_=0.47 with sleep medication prescription (108) r_G_=0.67 with nightmares (109) r_G_=0.24 with difficulty awakening (35)
Bipolar disorder	–	r_G_=0.11-0.19 with insomnia (31–33) r_G_=-0.05 or n.s. with being a morning person (32,33) r_G_=0.14-15 with daytime nap (32,33,103) r_G_=0.12-0.13 with excessive daytime sleepiness (32,33) r_G_=0.21-0.25 with long sleep duration, r_G_=-0.16 or n.s. with short sleep duration, r_G_=0.11 with continuous sleep duration (32,33,104) r_G_=0.31 with sleep medication prescription (108) r_G_=0.13 with difficulty awakening (35)
Schizophrenia	–	r_G_=0.18 or n.s. with insomnia (31–33) r_G_=-0.12 - -0.13 with being a morning person (32,33) r_G_=0.14-0.19 with daytime nap (32,33,103) r_G_=0.07-0.09 with excessive daytime sleepiness (32,33) r_G_=0.25-0.3 with long sleep duration, r_G_ with short sleep duration, r_G_=0.13-0.15 with continuous sleep duration (32,33,104) r_G_=-0.13 with sleep health scores (106) r_G_=0.25 with sleep medication prescription (108) r_G_=0.23 with difficulty awakening (35)
ADHD or ADHD symptoms	n.s. r_A_ with sleep problems in children (111), but r_A_ =0.49 in young adults (112)	r_G_=0.4-0.46 with insomnia (31,33) r_G_=0.19 with daytime nap (33) r_G_=0.16 with excessive daytime sleepiness (33) r_G_=0.38 with long sleep duration, r_G_=0.36-0.53 with short sleep duration (33,104) r_G_=-0.25 with sleep health scores (106) r_G_=0.3 with sleep medication prescription (108) r_G_=0.29 with RLS (110)
ASD	r_A_ =0.62 with difficulties initiating and maintaining sleep (113)	r_G_=-0.18 with being a morning person (33) r_G_=0.09 with daytime nap (33) r_G_=0.14 with excessive daytime sleepiness (33) r_G_=-0.17 with sleep health scores (106)
PTSD	n.s. r_A_ with sleep duration in adults (114)	r_G_=0.36-0.49 with insomnia (33,115) r_G_=-0.23 with being a morning person (115) r_G_=0.21-0.22 with daytime nap (33,115) r_G_=0.17 with excessive daytime sleepiness (33) r_G_=0.3-0.44 with long sleep duration, r_G_=0.44- 0.49 with short sleep duration (33,115) r_G_=0.36 with sleep medication prescription (108) r_G_=0.41 with nightmares (109) r_G_=0.23 with difficulty awakening (35)
Anorexia nervosa	–	r_G_=0.1 with insomnia (33) r_G_=-0.15 with daytime nap (33) r_G_=-0.11 with long sleep duration (33) r_G_=0.25 with sleep medication prescription (108)

**Table 2:** MR evidence of causal relationship between neuropsychiatric conditions and sleep.

Disorder	Sleep traits influence neuropsychiatric conditions	Neuropsychiatric conditions influence sleep traits
Depression or depressive symptoms	↑Insomnia (33,59–63) ↑ long or longer sleep duration (33,62) ↑ short sleep duration (33) ↑ daytime napping (33,59,62) ↑ OSA (64–68) but not in (62) ↑ narcolepsy (116) but not in (117)	↑Insomnia (32,33,69) ↑daytime napping (32,33) ↑long sleep duration (32,33) ↑short sleep duration (69) ↑ OSA (64,65,68)
Anxiety	↑Insomnia (33,60,118) ↓ being a morning person (33) ↑ narcolepsy (119)	–
Bipolar disorder	↑Insomnia (59,61) ↑daytime napping (33),	↑daytime napping (33) ↑long sleep duration (32)
Schizophrenia	↓ being a morning person (120) ↑ longer sleep duration (59,120) ↑daytime napping (120) ↑ narcolepsy (117)	↑daytime napping (32,33,120) ↓ being a morning person (32,120) ↑long sleep duration (32,120) ↓ short sleep duration (120)
ADHD	↑Insomnia (33) ↑short sleep duration (33) ↑daytime napping (33) timing of most active 10 hours measured by accelerometer (121)	↑Insomnia (33) ↑short sleep duration (33) ↑long sleep duration (33) ↑RLS (122)
ASD	↑ daytime napping (33) timing of most active 10 hours measured by accelerometer (121)	–
PTSD	↑Insomnia (33)	–
Anorexia nervosa	↑ being a morning person (123) ↓ daytime napping (33)	↑ being a morning person (123) ↑short sleep duration (33)
